# Selective Expansion of NKG2C+ Adaptive NK Cells Using K562 Cells Expressing HLA-E

**DOI:** 10.3390/ijms23169426

**Published:** 2022-08-20

**Authors:** Minh-Trang Thi Phan, Jinho Kim, Seung Kwon Koh, Yuree Lim, Hongbi Yu, Mijeong Lee, Jong-Min Lee, Eun-Suk Kang, Hyun-Young Kim, Sang-Ki Kim, Ilwoong Hwang, Duck Cho

**Affiliations:** 1 Cell and Gene Therapy Institute (CGTI), Samsung Medical Center, Seoul 06351, Korea; 2 Department of Health Sciences and Technology, Samsung Advanced Institute for Health Sciences and Technology, Sungkyunkwan University, Seoul 06355, Korea; 3 Department of Biopharmaceutical Convergence, Sungkyunkwan University, Suwon 16419, Korea; 4 Bio Research Center, Lugensci Co., Ltd., Bucheon 14556, Korea; 5 Department of Laboratory Medicine and Genetics, Samsung Medical Center, Sungkyunkwan University School of Medicine, Seoul 06351, Korea; 6 Department of Companion and Laboratory Animal Science, Kongju National University, Yesan 32439, Korea; 7 Department of Emergency Medicine, Soonchunhyang University Gumi Hospital, Gumi 39371, Korea

**Keywords:** adaptive NK cells, effective expansion, K562-HLA-E feeder cells, long-term persistence, single KIR NKG2C

## Abstract

Adaptive natural killer (NK) cells expressing self-specific inhibitory killer-cell immunoglobulin-like receptors (KIRs) can be expanded in vivo in response to human cytomegalovirus (HCMV) infection. Developing a method to preferentially expand this subset is essential for effective targeting of allogeneic cancer cells. A previous study developed an in vitro method to generate single KIR+ NK cells for enhanced targeting of the primary acute lymphoblastic leukemia cells; however, the expansion rate was quite low. Here, we present an effective expansion method using genetically modified K562-HLA-E feeder cells for long-term proliferation of adaptive NK cells displaying highly differentiated phenotype and comparable cytotoxicity, CD107a, and interferon-γ (IFN-γ) production. More importantly, our expansion method achieved more than a 10,000-fold expansion of adaptive NK cells after 6 weeks of culture, providing a high yield of alloreactive NK cells for cell therapy against cancer.

## 1. Introduction

Natural killer (NK) cells are innate lymphocytes that kill virus-infected, stressed, or transformed cells [1,2]. NK cells are a component of the innate immune system; however, recent studies have revealed a substantial and long-lasting increase in the NKG2C+ NK cell subset as an imprint of human cytomegalovirus (HCMV) infection. These “adaptive NK cells” have been shown to display adaptive immune features [3,4,5,6] and a differentiated phenotype, including lower expressions of signaling adapter FcεRIγ, signaling intermediate Syk, and transcription factor PLZF [7] and higher expressions of CD57, CD2, and granzyme B [8]. An attractive characteristic of adaptive NK cells is their preferred expression of inhibitory killer-cell immunoglobulin-like receptors (KIRs) specific for self-human leukocyte antigen (HLA) class I molecules [9,10,11].

NK cells expressing a single type of KIR are highly cytotoxic against HLA class I-mismatched allogeneic acute myeloid leukemia blasts [12,13]. Moreover, selectively expanded adaptive NK cells with a single self-specific KIR efficiently kill acute lymphoblastic leukemia cells with HLA-mismatched targets [14]. In addition, adaptive NK cells play important roles in the treatment of multiple myeloma using monoclonal antibodies, such as daratumumab, by enhancing anti-myeloma antibody-dependent cellular cytotoxicity (ADCC) and exerting robust daratumumab-mediated effector functions [15,16]. Therefore, it would be beneficial to develop ex vivo expansion protocols for the selective expansion of single positive KIR subsets in order to increase the number of alloreactive NK cells available for infusion.

Previous studies have indicated that the presence of the non-classic major histocompatibility complex class I molecule HLA-E (cognate ligand for NKG2C and NKG2A) is necessary for the expansion of adaptive NK cells [9,17]. Liu et al., first reported a robust and scalable protocol using 721.221 feeder cells expressing HLA-E for ex vivo generation and expansion of adaptive NK cells for use in cell therapy against pediatric acute lymphoblastic leukemia [14]. However, the expansion rate of these adaptive NKG2C+ NK cells was minimal.

Our group has examined the frequency of occurrence of FcεRIγ-deficient NK cells (termed g-NK cells), which appear in HCMV-positive donors and most often express the activating receptor NKG2C [18] in cord blood and adult blood samples from the Korean population [19]. It has been reported that g-NK cells, though not identical to adaptive NKG2C+ NK cells, also exhibit overlapping adaptive features such as clonal expansion, enhanced response to CD16 cross-linking, and long-term persistence [15,18,20,21].

We have studied and developed effective protocols for the ex vivo expansion of NK cells from peripheral blood mononuclear cells (PBMCs) using both conventional K562 cells and genetically modified K562 cells expressing cofactor OX40L and cytokines mbIL-18 and -21 [22,23,24,25]. We also comparatively analyzed the effects that various culture media and supplements exerted on the expansion of NK cells obtained from cord blood samples [26].

Based on these results, we reasoned that genetically modified K562 cells expressing HLA-E would perhaps function as effective feeder cells for the improvement of the expansion rate of adaptive NKG2C+ NK cells. To validate this in the present study, we compared conventional K562- and K562-HLA-E-based culture methods and investigated the phenotypic and functional effects exerted by K562 cells expressing HLA-E on the expansion of adaptive NKG2C+ NK cells.

## 2. Results

### 2.1. Generation of Genetically Engineered K562 Cells Expressing HLA-E

**Figure 1:**
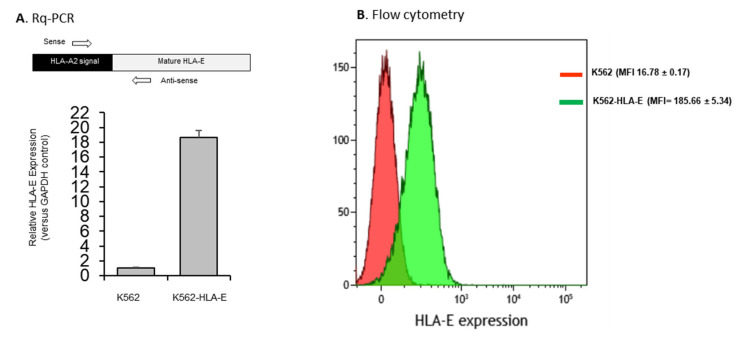
HLA-E expression of genetically engineered K562 cells. (**A**) mRNA expression by K562 and K562-HLA-E cells were determined using real-time quantitative polymerase chain reaction (Rq-PCR) with glyceraldehyde-3-phosphate dehydrogenase (GAPDH) as control. (**B**) HLA-E surface expression was analyzed using fluorescence-activated cell sorting (FACS) with anti-human HLA-E (clone 3D12) in K562 (red) and K562-HLA-E (green) cells.

HLA-E expression in K562-HLA-E cells was confirmed at both the transcriptional and translational levels. Real-time quantitative polymerase chain reaction (Rq-PCR) analysis performed using the total RNA isolated from K562 and K562-HLA-E cell lysates showed a significantly higher (18.7-fold) level of HLA-E mRNA expression in K562-HLA-E cells than in K562 control cells (Figure 1A). This was further confirmed through the flow cytometric analyses of K562-HLA-E and conventional K562 cells (Figure 1B).

### 2.2. K562-HLA-E Feeder Cells Enhanced the Expansion of NK Cells

**Figure 2:**
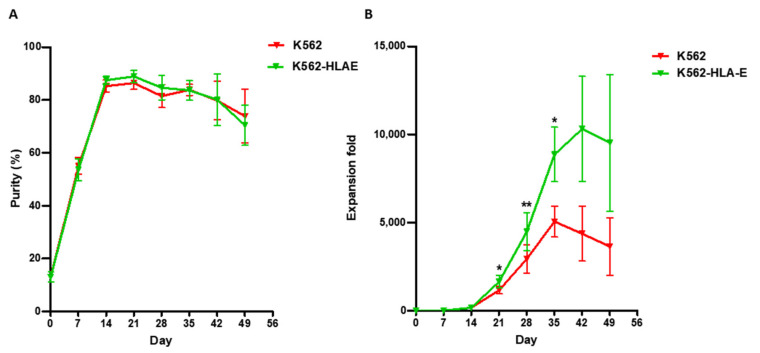
Purity and fold expansion of expanded natural killer (NK) cells co-cultured with K562 and K562-HLA-E. feeder cells. Freshly isolated peripheral blood mononuclear cells (PBMCs) were co-cultured with γ-irradiated K562 (red) and K562-HLA-E (green) feeder cells in the presence of IL-2 (10 U/mL) at day 0 and IL-2 (100 U/mL) and IL-15 (5 ng/mL) after 1 week. The medium was replaced every 2–3 days along with fresh cytokines. (**A**) Purity of expanded NK cells was determined by flow cytometry using fluorescein isothio-cyanate (FITC)-conjugated anti-human CD3 and phycoerythrin (PE)-Cy5-conjugated anti-human CD56. (**B**) Fold expansion of NK cells in the K562 vs. K562-HLA-E added groups was significantly different from day 21 of culture. All data are shown as the mean ± SEM (*n* = 9 independent experiments; * *p* < 0.05; ** *p* < 0.01).

To study the effect exerted by K562-HLA-E feeder cells on NK cell expansion rate, we compared the purity and fold expansion of the NK cells cultured on K562 and K562-HLA-E cells. Similar purity was observed in the eNK cells after 7 weeks, irrespective of whether they were cultured with K562 or K562-HLA-E cells (Figure 2A). However, after 3–5 weeks of culture, an increased NK cell expansion fold was observed in the cultures with K562-HLA-E cells than in cultures with K562 cells (Figure 2B). Mean expansion (range) was 1162 (435.1–1892.67), 2939 (1299.7–9170.33), and 5051 (2443.22–8041.57) in the K562 group and 1640 (359.78–3072), 4475 (1632.43–12748.8), and 8856 (4063.49–16527) in K562-HLA-E group.

### 2.3. Selective Expansion of Single KIR+ Cells Co-Cultured with PBMCs on K562-HLA-E Feeder Cells

**Figure 3:**
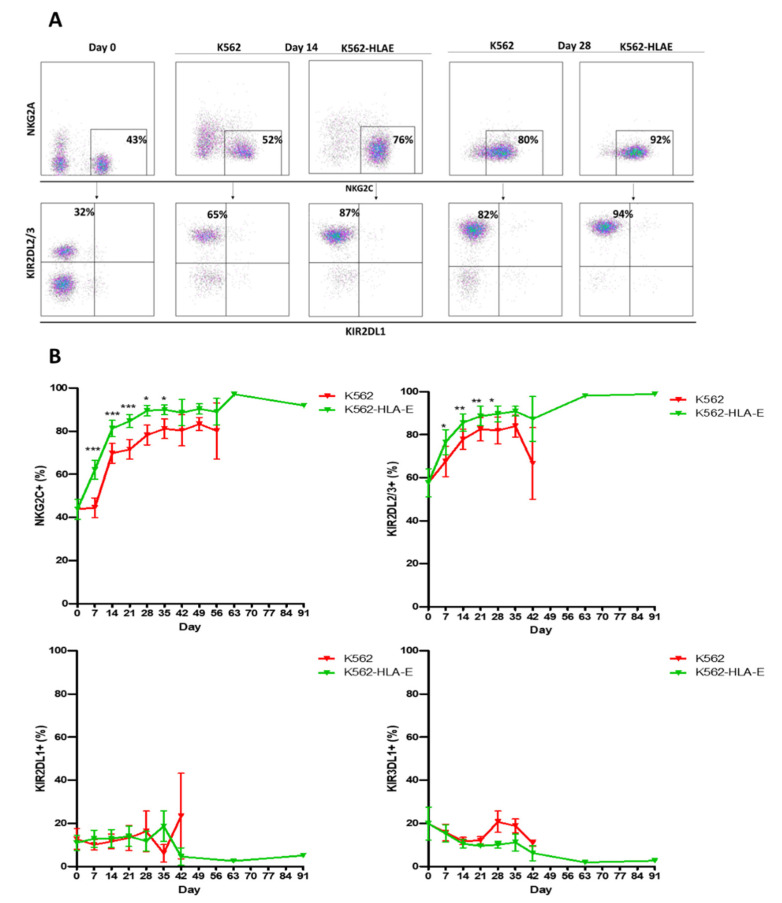
Preferred expansion high frequencies of NKG2C+ natural killer (NK) cells expressing single KIR2DL2/3 by K562-HLA-E in C1C1 donors. (**A**) Representative fluorescence-activated cell sorting (FACS) plot showing the frequency of NKG2C, NKG2A, KIR2DL1, and KIR2DL2/3 at days 0, 14, and 28 of culture. (**B**) Percentage expression of NKG2C, KIR2DL2/3, KIR2DL1, and KIR3DL1 on adaptive NK cells (K562-HLA-E) and conventional NK cells (K562) during culture. All data are shown as mean ± SEM (*n* = 9 independent experiments; * *p* < 0.05; ** *p* < 0.01; *** *p* < 0.001).

To test whether K562-HLA-E feeder cells were responsible for promoting adaptive NK cell expansion, NKG2C+ cells obtained from 16 healthy donors were cultured on irradiated K562 and K562-HLA-E feeder cells, and the KIR expression was compared. The clinical characteristics of the donors are presented in Table 1. Selective expansion of NKG2C+ cells with self-specific KIR2DL2/3 expression was observed in cells obtained from C1C1 donors cultured with K562-HLA-E cells rather than with K562 cells (Figure 3A). Long-term survival (over 3 months) was observed in the adaptive NKG2C+ cells with single KIR2DL2/3+ expression generated with K562-HLA-E cells rather than in conventional NK cells generated with K562 cells (Figure 3B). Enhanced NKG2C+ population and self-specific KIR were also observed in cells obtained from C1C2 and C2C2 donors (Supplementary Figure S1).

### 2.4. Phenotypic Characteristics of eNK Cells Using K562 and K562-HLA-E as Feeder Cells

**Figure 4:**
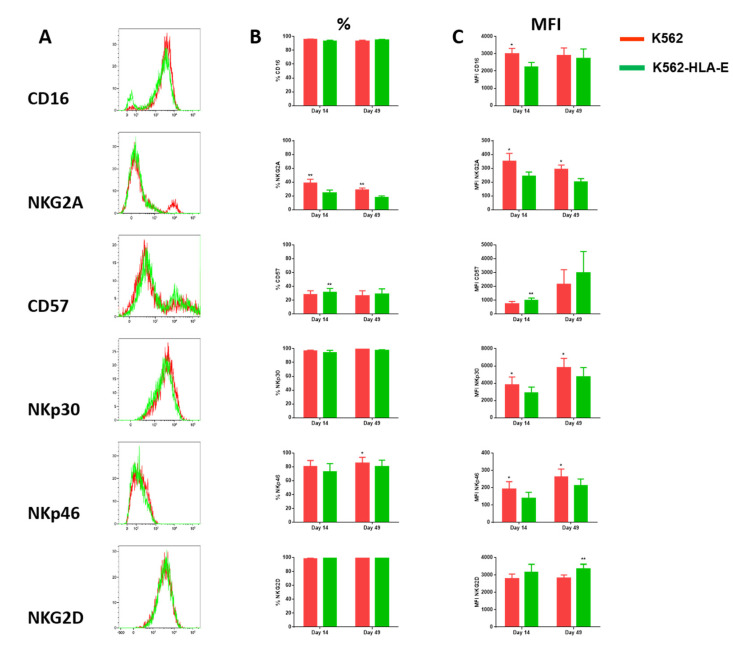
Comparison of receptor expression levels of expanded natural killer (NK) cells using K562 and K562-HLA-E cells. (**A**) Expression of the indicated surface receptors (CD16, NKG2A, CD57, NKp30, NKp46, and NKG2D) on expanded NK cells. Representative histograms are shown in the NK cell subset from the same donor on conventional (K562, red) and adaptive (K562-HLA-E, green) NK cells at day 14 of culture. (**B**) Differences in the expression levels (percentage of positive) of CD16, NKG2A, CD57, NKp30, NKp46, and NKG2D of conventional (K562) and adaptive (K562-HLA-E) NK cells on day 14 and day 49 of culture (*n* = 7–9 donors). (**C**) Differences in the expression levels (mean fluorescence intensity MFI)) of CD16, NKG2A, CD57, NKp30, NKp46, and NKG2D of conventional (K562) and adaptive (K562-HLA-E) NK cells on day 14 and day 49 of culture. All data are shown as mean ± SEM (*n* = 7–9 independent experiments; * *p* < 0.05; ** *p* < 0.01).

Comparisons of the phenotypic characteristics of NK cells expanded with K562 and K562-HLA-E cells were made on days 14 and 49 of culture. The eNK cells co-cultured with K562-HLA-E cells exhibited “adaptive” receptor features such as reduced CD16 (day 14) and NKG2A (days 14 and 49) expression, increased CD57 (day 14) expression, and slightly reduced NKp30 and NKp46 (day 14 and day 49) expression. Interestingly, eNK cells showed slightly higher NKG2D expression when expanded with K562-HLA-E cells than with K562 cells (Figure 4).

### 2.5. Expression of FcεRIγ and NKG2C in NK Cells during Expansion

**Figure 5:**
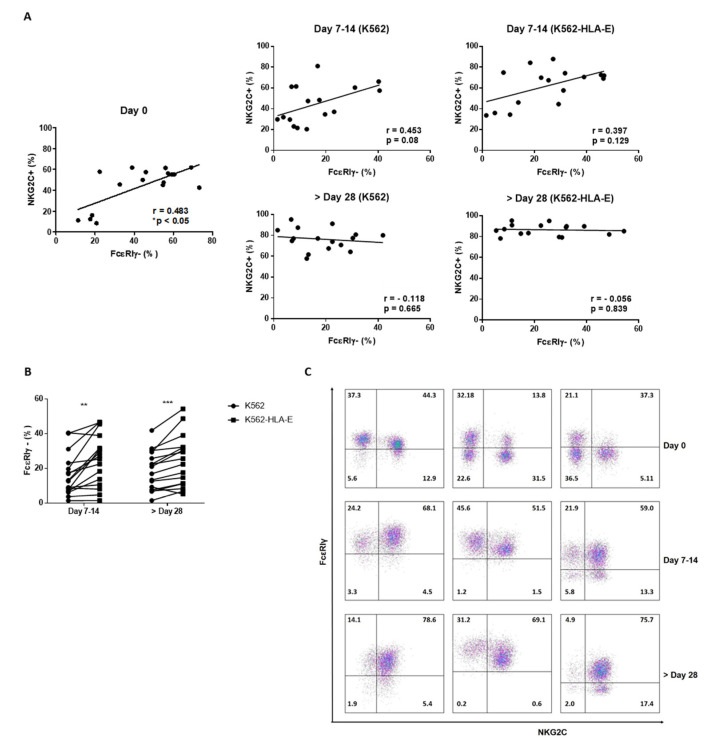
The frequency of FcεRIγ and NKG2C in natural killer (NK) cells during expansion. (**A**) Correlation of the frequency of FcεRIγ- NK cells and NKG2C+ NK cells before and following NK cell expansion (*n* = 16 donors). (**B**) Comparison of proportions of FcεRIγ- expression between NK cells expanded with K562 and K562-HLA-E cells. (**C**) Representative flow cytometry plots of FcεRIγ and NKG2C of three donors during NK cell expansion with K562-HLA-E cells. All data are shown as mean ± SEM (*n* = 16 independent experiments; ** *p* < 0.01; *** *p* < 0.001).

Along with a high frequency of NKG2C expression, a deficiency of FcεRIγ expression is a characteristic feature of adaptive NK cells. Therefore, we investigated whether the K562-HLA-E-based eNK cells preferentially boosted the NKG2C+ FcεRIγ- NK cell population. Before expansion, the percentage of FcεRIγ- NK cells was significantly correlated with the percentage of NKG2C+ NK cells (Figure 5A), although NKC2C+ cells were not always FcεRIγ- and vice versa (Figure 5C). Surprisingly, an increased expression of FcεRIγ+ NK cells was observed after expansion, resulting in the NKG2C+ FcεRIγ+ population being the most predominant among the four populations (NKC2C+ FcεRIγ+, NKC2C+ FcεRIγ-, NKC2C- FcεRIγ+, and NKC2C- FcεRIγ-). There was no significant correlation between NKC2C+ and FcεRIγ- cells in the expanded NK cells either after 1–2 weeks or in the late stage of culture (Figure 5A). Although the reason behind the increase in FcεRIγ+ population during culture remains elusive, we noted that the K562-HLA-E-based expanded NK cells retained a greater portion of the FcεRIγ- population than did the K562-based eNK cells (Figure 5B).

### 2.6. Function of In Vitro Generated eNK Cells Using K562 and K562-HLA-E Feeder Cells

**Figure 6:**
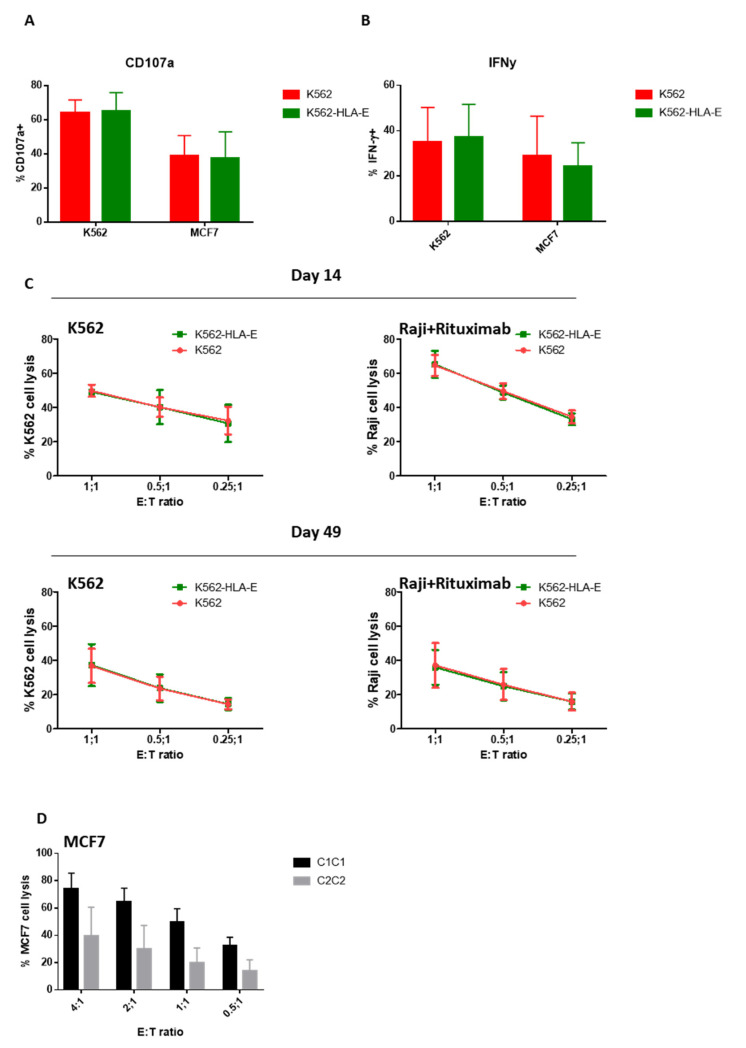
Function of expanded natural killer (eNK) cells using K562 and K562-HLA-E cells. Surface expression of CD107a (**A**) and intracellular expression of IFN-γ (**B**) of eNK cells were measured by incubation of eNK cells with K562 cells at an effector-to-target (E:T) ratio of 1:1 for 5 h followed by evaluation using flow cytometry. Bar graphs show the percentage of eNK cells for degranulation (CD107a) and IFN-γ (error bars, mean ± SD). (**C**) The cytotoxicity of eNK cells on days 14 and 49 toward K562 cells, and ADCC of eNK cells against rituximab-coated Raji cells were measured by carboxyfluorescein succinimidyl ester (CFSE)-based flow cytometry assay at E:T ratios of 1:1, 0.5:1, and 0.25:1 for 4 h. Results presented are the mean of 6 donors and error bars represent the mean ± SD. (**D**) The cytotoxicity of K562-HLA-E based eNK cells of C1C1 and C2C2 donors against MC7 (C2C2) target cells.

To investigate the difference in the functionality of eNK cells when K562 and K562-HLA-E cells were used, we measured CD107a degranulation and IFN-γ production using K562 and MCF7 as target cells. Similar levels of CD107a degranulation and IFN-γ production were observed in eNK cells stimulated with K562 or MCF7 target cells irrespective of whether they were generated with K562-HLA-E or K562 cells (Figure 6A,B).

We further examined cytotoxic effector functions using a (carboxyfluorescein succinimidyl ester) CFSE-based killing assay against K562 target cells and Raji cells coated with rituximab because ADCC is generally considered an essential function of adaptive NK cells [18,20,27]. However, no significant differences were found between NK cells expanded with K562 and K562-HLA-E cells at various E:T ratios on day 14 or the latter stage (day 49) of culture (Figure 6C).

A trend of higher cytotoxicity was observed against HLA-mismatched MCF7 target cells (C2C2) in the eNK cells that used K562-HLA-E cells from C1C1 donors compared with those from C2C2 donors (Figure 6D), although no significant differences were detected.

## 3. Discussion

The expansion rate of NK cells is affected by various factors such as the source of NK cells, cytokines, feeder cells, media, and sample status (fresh vs. cryopreserved) [26,28,29,30]. Liu et al. developed an adaptive NK cell expansion method using NK cells isolated from thawed PBMCs co-cultured with HLA-transfected 721.221 feeder cells in the presence of IL-15 (100 ng/mL), which led to a 2.4-fold increase after 14 days of culture [14]. In the present study, we achieved a 169 ± 94-fold expansion after 14 days and a 10,311 ± 6676-fold expansion after 42 days of culture of NK cells from PBMCs co-cultured with γ-irradiated K562-HLA-E feeder cells in the presence of IL-2 and IL-15. The huge difference in the outcomes of NK cell expansion in the two studies might be attributed to the type of feeder cells used and the source of NK cells (we used fresh instead of cryopreserved samples). In addition, the combination of IL-2 and IL-15 in our method may have induced a higher NK cell proliferation than IL-15 alone.

The presence of HLA-E, which serves as a cognate ligand for NKG2C, is necessary for the expansion of adaptive NKG2C+ NK cells [9,14,17,31], and these adaptive NK cells survived longer than did the conventional NK cells [32]. However, the stability and efficacy of the expansion methods using HLA-E-transfected cells remain to be determined. Both protocols (one utilizing HLA-E-transfected 721.221 cells and the other utilizing K562 cells expressing the HLA-E) showed selective expansion and long-term persistence of NKG2C+ single KIR+ NK cells. However, cell survival was markedly prolonged in the cultures with K562-HLA-E feeder cells than with K562 cells; in fact, these cells were viable after 3 months of culture (Figure 3). Previous studies and our data suggest that HLA-E expressed on feeder cells plays a critical role in the expansion of adaptive NKG2C+ NK cells and contributes to prolonged cell survival in the presence of cytokines.

To further characterize the expanded adaptive NK cells, we performed flow cytometric analysis of various NK cell receptors. Consistent with previous findings [32,33,34], the adaptive NK cells generated by us showed reduced CD16, NKG2A, NKp30, and NKp46 expression and increased CD57 expression compared to conventional NK cells.

Traditionally, adaptive NK cells have an FcεRIγ-deficient phenotype [20,35,36]. However, in our study, the eNK cells, irrespective of whether they were co-cultured with K562 or K562-HLA-E feeder cells, showed an increase in the percentage of FcεRIγ+ cells during culture, even though K562-HLA-E-based eNK cells preserved a higher portion of FcεRIγ- population than K562-based eNK cells. Hence, the continued supplementation of cytokines (IL-2 and IL-15) during NK cell expansion can partially alter the phenotype of the adaptive NK cells.

Despite varied expression of several receptors, the expanded adaptive NK cells remained highly functional and showed similar cytotoxicity and CD107a degranulation levels against K562 cells, as did conventional NK cells. In contrast to previous studies [15,16], K562-HLA-E-expanded adaptive NK cells did not show enhanced ADCC function and IFN-γ production compared with that of K562-expanded conventional NK cells. Presumably, this might be due to the highly activated eNK cells (in both the K562 and K562-HLA-E systems) expressing high levels of activating receptors (NKG2C, NCR, and NKG2D) and low levels of CD16 in our culture system because adaptive NK cells preferentially produce IFN-γ and other cytokines in response to FcγRIIIa triggering [34]. Additional studies are required to explore ways to maintain a stable CD16 expression during the culture period.

Therefore, this expansion process can influence the adaptive NK cell phenotype and functional features. Further studies comparing isolated adaptive and expanded adaptive NK cells are needed to understand the potential mechanism underlying this alteration.

Moreover, our data showed that expanded NKG2C+ NK cells could persist for up to 13 weeks when cultured with genetically modified K562 cells expressing HLA-E. Even after long-term culture, they remained functional and expressed the same patterns of surface markers, unlike adaptive NK cells after short-term culture (Figure 4B,C and Figure 6C). Similarly, a previous study showed that adoptively transferred adaptive NK cells exhibited enhanced persistence in vivo than did conventional NK cells [15]. In addition, Zhang et al. reported that adaptive NK cells persisted stably for over 9 months in an individual while showing higher expression levels of the anti-apoptotic molecule bcl-2, suggesting that adaptive NK cells might be more resistant to apoptosis [18]. Taken together, the selective expansion of adaptive NK cells with our genetically modified K562 feeder cells provides a promising clinical option to treat cases of refractory or relapsing malignancies, which require long-term persistence of effector cells.

Our study had several limitations. Although K562-HLA-E cells induced a higher NK cell expansion rate than in the previous study, a direct comparison with other feeder cells (including HLA-E-transfected 721.221 cells) in the same culture system should be verified by future studies. In addition, the combination of HLA-E, other co-factors, and cytokines (such as IL-12, IL-18, and IL-21) required to optimize the expansion rate of adaptive NK cells needs investigating. Another limitation of our study is that the frequency of the HLA-C2 ligand is much lower in the Korean population than in Caucasians (3.2%) [37], and the expanded adaptive NKG2C+ NK cells in the present study have a predominant clonal expression pattern of KIR2DL2/3 (from C1C1 donors). Consistent with previously reported data [14], expanded adaptive NK cells from C1C1 donors showed the effective killing of mismatched MCF7 (C2C2) target cells, reaching 74.2 ± 19.6% target cell lysis at an E:T = 4:1 ratio (Figure 6D), compared with that of C2C2 donors (39.7 ± 36.1%). The cytotoxicity of expanded adaptive NK cells from C2C2 donors in our study was quite high, in contrast to the negligible killing in the KIR-HLA-matched set of the previous study [14]. This might be associated with the small sample size of C2C2 donors in this study, and the fact that two-thirds of C2C2 donors had a high proportion of KIR2DL1+ KIR2DL2/3+ NK cells instead of KIR2DL1+ NK cells (Supplementary Figure S1).

Further studies with larger patient populations are required to confirm the influence of KIR ligand status on the generated specific adaptive NK cells (with single KIR expression) for the clinical targeting of cancer across HLA barriers in cell therapy.

In conclusion, we successfully developed a remarkable ex vivo expansion method using K562-HLA-E feeder cells to mimic CMV triggers and selectively expand long-lived adaptive NKG2C+ NK cells expressing self-specific KIR. Furthermore, long-lived expanded adaptive NK cells demonstrated sustained cytolytic activity. Further studies are needed to uncover the relevant aspects of expanded adaptive NK biology in order to optimize their clinical applications.

## 4. Materials and Methods

### 4.1. Samples

Peripheral blood samples from 16 healthy donors were collected in heparinized tubes. Informed consent was obtained prior to study participation and blood sample collection. This study was approved by the Institutional Review Board of Samsung Medical Center, Seoul, Korea (IRB No. SMC 2020).

### 4.2. Cells and Culture

K562 (human myelogenous leukemia cell line) and Raji (human Burkitt’s lymphoma cell line) cells were obtained from the American Type Culture Collection (Manassas, VA, USA). The cells were cultured in RPMI 1640 medium supplemented with 10% heat-inactivated fetal bovine serum (FBS) (Gibco, Grand Island, NY, USA), penicillin (100 units/mL), and streptomycin (100 μg/mL) (Invitrogen, Carlsbad, CA, USA) at 37 °C in a humidified 5% CO_2_ incubator.

### 4.3. Generation of Genetically Engineered K562 Expressing HLA-E

The hybrid HLA-E sequence was synthesized after the signal domain of HLA-A2 was fused with the mature HLA-E sequence, as reported previously [38]. As the sequence of mature–hybrid HLA-E is the same as that of HLA-E, it is referred to as HLA-E in the following text. The sequences were verified using DNA sequencing after gene synthesis. The synthetic HLA-E sequence was then ligated between the XbaI and EcoRI sites of the pCDH-CMV-EF1-GFP vector (System Biosciences, Palo Alto, CA, USA) to yield pCDH-CMV-HLA-E-EF1-GFP. Then 293FT cells were co-transfected with the HLA-E-transfer plasmid and lentiviral packaging mixture (pLP1, pLP2, and pLP/VSVG; Thermo Scientific, Waltham, MA, USA) to produce lenti-HLA-E. Transfections were performed using E-fectin Plus (Lugen Sci Co. Ltd., Bucheon, South Korea) according to the manufacturer’s instructions. At 48 h post-transfection, the viral supernatant was collected, and the viral titer was determined using a Lenti-X qRT-PCR titration kit (Takara Bio Inc., Otsu, Shiga, Japan). To produce genetically engineered K562 cells expressing HLA-E, K562 cells were seeded into 6-well plates at 3 × 10^5^ cells/well and then transduced with the lentiviral supernatant for 24 h in the presence of polybrene (8 μg/mL; Sigma-Aldrich, St. Louis, MO, USA) at a multiplicity of infection of 30. After 24 h of incubation, the virus-containing medium was removed and replaced with 2 mL of fresh culture medium. When cell confluency was over 90%, the cell culture was expanded to a T-25 flask. The transduction efficiency was evaluated daily using an inverted fluorescence microscope. Two weeks after transduction, GFP-positive cells were sorted using BD FACSAria TM III and maintained in RPMI 1640 with 10% FBS.

### 4.4. Real-Time Quantitative Polymerase Chain Reaction (Rq-PCR) Detection of HLA-E mRNA

Total RNA for mRNA Rq-PCR analysis was isolated using an RNeasy Mini Kit (Qiagen, Venlo, The Netherlands) according to the manufacturer’s instructions and quantified using an IMPLEN nanophotometer P330 (IMPLEN, Munich, Germany). Isolated RNAs were converted to cDNA using the QuantiTect Reverse Transcription Kit (Qiagen). All PCRs were performed using a QuantiTect SYBR Green PCR Kit (Qiagen) and Rotor-Gene Q (Qiagen) in standard 20 µL reactions. A sense primer (5′-ACCCTCGTCCTGCTACTCTC-3′) targeting the signal sequence of HLA-A2 and an antisense primer (5′-CCACGTAGCCCACAGAGATG-3′) targeting the mature sequence of HLA-E were used to exclusively amplify the HLA-E gene. Glyceraldehyde-3-phosphate dehydrogenase (GAPDH) was amplified using the sense primer 5′-ACATCGCTCAGACACCATG-3′ and antisense primer 5′-TGTAGTTGAGGTCAATGAAGGG-3′. All the samples were processed in triplicate to increase the reliability of the results. Each assay was performed using positive and negative controls. The threshold cycle (Ct) value for each gene was measured for each sample. The Ct value of GAPDH was used as an endogenous reference for normalization. The values obtained were normalized to those of the negative control and expressed as fold-changes.

### 4.5. Cytokines and Antibodies

Recombinant human interleukin (IL)-2 and IL-15 (PeproTech, Rocky Hill, NJ, USA) were used to expand NK cells. Phycoerythrin (PE)-conjugated anti-human HLA-E (clone 3D12) (BioLegend, San Diego, CA, USA) was used to measure HLA-E expression on generically modified K562-HLA-E during culture. Allophycocyanin (APC)-Cy7-conjugated anti-human CD3, PE-Cy7-conjugated anti-human CD56, pacific blue-conjugated anti-human CD16, fluorescein isothiocyanate (FITC)-conjugated anti-human CD57, pacific blue-conjugated anti-human NKp46, PE-conjugated anti-human NKp30, peridinin chlorophyll protein complex (PerCP)-conjugated anti-human NKG2D (eBioscience, San Diego, CA, USA), FITC-conjugated anti-human CD69, APC-conjugated anti-human DNAM-1 (BD Biosciences, San Jose, CA, USA), APC-conjugated anti-human NKG2A, PE-conjugated anti-human NKG2C, PerCP-conjugated anti-human KIR2DL1 (R & D Systems, Minneapolis, MN, USA), FITC-conjugated anti-human KIR2DL2/L3, and BV421-conjugated anti-human KIR3DL1 (BioLegend) were used to evaluate purity and surface expression of NK cell receptors. The PE-conjugated anti-human CD107a mAb was used as a surrogate marker for quantifying degranulation. BV421-conjugated anti-human interferon (IFN)-γ and FITC-conjugated anti-FcεRIγ subunit (FcɛRγ) (Millipore, CA, USA) were used for intracellular staining.

### 4.6. Ex vivo NK Cell Expansion Using K562 and K562-HLA-E Feeder Cells

Human PBMCs were isolated from healthy adult donors using density-gradient centrifugation with Ficoll-Hypaque (d = 1.077, LymphoprepTM; Axis-Shield, Oslo, Norway) and washed twice with phosphate-buffered saline (Welgene, Gyeongsan-si, Korea). PBMCs were co-cultured with irradiated (100 Gy) K562 and K562-HLA-E cells in a 24-well plate with RPMI 1640 medium (10% FBS, penicillin (100 U/mL), streptomycin (100 µg/mL), and L-glutamine (4 mmol/L)) containing recombinant human IL-2 (10 U/mL). After day 7, the IL-2 concentration was increased from 10 to 100 U/mL, and soluble IL-15 (5 ng/mL) was added to the medium. The irradiated feeder cells were re-stimulated on days 7 and 14. The medium was replaced every 2–3 days. The expansion rate of NK cells is presented as the “expansion fold”, which was determined by dividing the absolute number of NK cells at time points of interest by the respective number on day 0.

### 4.7. Surface and Intracellular Staining Using Flow Cytometry

HLA-E expression on conventional K562 and K562-HLA-E cells and the expression of NK cell receptors on expanded NK (eNK) cells were examined by staining with an appropriate combination of fluorochrome-conjugated monoclonal antibodies (HLA-E, CD3, CD56, CD16, CD57, NKp30, NKp46, DNAM-1, NKG2A, NKG2C, NKG2D, KIR2DL1, KIR2DL2/3, and KIR3DL1) for 15 min in the dark. To detect signaling adaptors, the cells were fixed, permeabilized, and stained with FITC-conjugated anti-FcɛRγ. After washing with fluorescence-activated cell sorting (FACS) buffer, stained cells were acquired using a FACS Verse instrument (BD Biosciences, San Jose, CA, USA).

To measure IFN-γ production, eNK cells were incubated in a 96-well U-bottom plate with K562 cells (effector-to-target ratio [E:T] = 1:1) in the presence of brefeldin A (BD Biosciences) at 37 °C and 5% CO_2_ for 5 h. Cells were then harvested, washed with FACS, and stained with anti-human CD3 and CD56 monoclonal antibodies (mAbs) for 20 min on ice. After washing, fixation, and permeabilization, NK cells were further stained with PE-conjugated anti-human IFN-γ mAb on ice for 30 min, washed, and analyzed using FACS Verse and Kaluza 1.3 (Beckman Coulter, Brea, CA, USA).

### 4.8. CD107a Degranulation

The eNK cells (2 × 10^5^) were incubated with or without 2 × 10^5^ K562 cells in a 96-well U-bottom plate in the presence of PE-conjugated anti-human CD107a (5 μL). Monensin and brefeldin A were added after 1 h, and the plate was incubated for an additional 4 h. NK cells were stained with anti-human CD3 and CD56 mAbs before acquisition.

### 4.9. Cytotoxicity and ADCC Assays

The cytotoxicity of eNK cells against target cells (K562, Raji, and MCF7) was measured using a CFSE-based assay for 4 h, as previously described [22,23]. Target cells were stained with CFSE (0.5 μM) in FACS buffer for 10 min at 37 °C. For the cytotoxicity assay, CFSE-stained K562 cells were incubated in a 96-well U-bottom plate with eNK cells at E:T ratios of 1:1, 0.5:1, and 0.25:1 for 4 h at 37 °C and 5% CO_2_. To perform the ADCC assay, CFSE-stained Raji cells were incubated with rituximab (1 μg/mL) and incubated with eNK cells at the same E:T ratios used for the cytotoxicity assay. The plates were centrifuged at 1500 rpm for 3 min and then incubated at 37 °C and 5% CO_2_ for 4 h. The mixed cells were transferred to FACS tubes after incubation and 1 μL of PI (1 mg/mL) (Invitrogen) was added to each tube. The cells were acquired on a FACS Verse and analyzed using the Kaluza software 1.3 (Beckman Coulter, Brea, CA, USA).

### 4.10. HLA-C Genotyping and HCMV Serology

HLA-C1 and HLA-C2 levels were determined by PCR amplification with sequence-specific primers (PCR-SSP) and low resolution using the HLA-C SSP PCR kit (BioSewoom, Seoul, Korea).

HCMV serology was determined using a BioMerieux VIDAS kit (VIDAS CMV IgG kit, VIDAS CMV IgM kit, Marcy-l’Étoile, France), and the cut-off value of seropositivity was ≥6 with CMVG and ≥0.90 with CMVM.

### 4.11. Statistical Analysis

Statistical analysis was performed using GraphPad Prism 5 (GraphPad Software, San Diego, CA, USA). The Mann–Whitney U-test (non-parametric) was used to analyze specific sample pairs for significant differences. All tests were two-tailed, and differences were considered statistically significant at *p* < 0.05.

## Figures and Tables

**Table 1:** Donor characteristics of cytomegalovirus (CMV) serology and human leukocyte antigen-C (HLA-C) genotyping.

CMVG	CMVM	KIR2DL2/3	KIR2DL1	KIR3DL1	HLA-C Genotyping
Positive	Negative	68.7	10.7	3.87	C1C1
Positive	Negative	61.9	49.1	7.53	C1C2
Positive	Negative	68.9	4.5	71.32	C1C1
Positive	Negative	79.3	3.3	5.14	C1C1
Positive	Negative	83.2	29.3	0.1	C1C1
Positive	Negative	45.9	10.4	1.97	C1C1
Positive	Negative	83.9	17.9	5.25	C1C1
Positive	Negative	36.7	87.4	6.77	C1C2
Positive	Negative	47.6	82.9	37.88	C1C2
Positive	Negative	96.4	1.0	1.51	C1C1
Positive	Negative	64.6	29.9	5.94	C1C2
Positive	Negative	78.2	73.3	10.84	C2C2
Positive	Negative	75.7	10.7	4.01	C1C1
Positive	Negative	43.0	15.3	24.94	C1C1
Positive	Negative	18.8	84.0	0.9	C2C2
Positive	Negative	52.2	86.5	62.55	C2C2

## Data Availability

Not applicable.

## Supplementary Materials

The supporting information can be downloaded at: https://www.mdpi.com/article/10.3390/ijms23169426/s1.
