# Supplementary material for: Selective Expansion of NKG2C+ Adaptive NK Cells Using K562 Cells Expressing HLA-E
Source: Int J Mol Sci. 2022 Aug 20;23(16):9426. doi: 10.3390/ijms23169426 (PMC9409060; doi:10.3390/ijms23169426)
Supplement: Supplementary file 1 [file ijms-23-09426-s001.zip › ijms-1863087-supplementary.pdf]

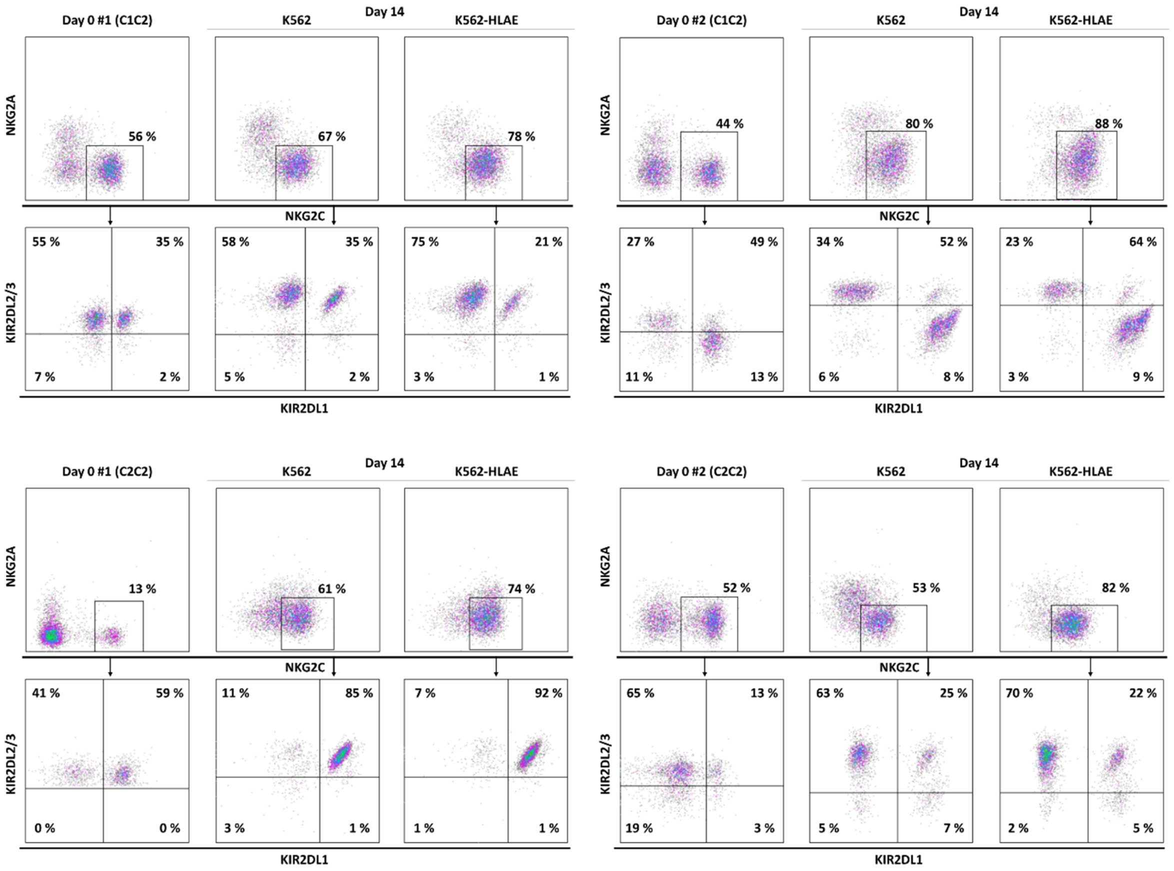

Supplementary Figure S1. Expansion of NK cells from C1C2 and C2C2 donors.
Representative FACS dot plot showing the frequency of NKG2C, NKG2A, KIR2DL1, KIR2DL2/3 in two C1C2 and two C2C2 donors before and after expansion with K562 or K562-HLA-E feeder cells
